# MaizeGDB: The Maize Model Organism Database for Basic, Translational, and Applied Research

**DOI:** 10.1155/2008/496957

**Published:** 2008-08-20

**Authors:** Carolyn J. Lawrence, Lisa C. Harper, Mary L. Schaeffer, Taner Z. Sen, Trent E. Seigfried, Darwin A. Campbell

**Affiliations:** ^1^USDA-ARS, Corn Insects and Crop Genetics Research Unit, Ames, IA 50011, USA; ^2^Department of Genetics, Development and Cell Biology, Iowa State University, Ames, IA 50011, USA; ^3^Department of Agronomy, Iowa State University, Ames, IA 50011, USA; ^4^USDA-ARS, Plant Gene Expression Center, 800 Buchanan Street, Albany, CA 94710, USA; ^5^Department of Molecular and Biology, University of California Berkeley, Berkeley, CA 94720, USA; ^6^USDA-ARS, Plant Genetics Research Unit, Columbia, MO 65211, USA; ^7^Division of Plant Sciences, University of Missouri Columbia, Columbia, MO 65211, USA

## Abstract

In 2001 maize became the number one production crop in the world with the Food and Agriculture Organization of the United Nations reporting over 614 million tonnes produced. Its success is due to the high productivity per acre in tandem with a wide variety of commercial uses. Not only is maize an excellent source of food, feed, and fuel, but also its by-products are used in the production of various commercial products. Maize's unparalleled success in agriculture stems from basic research, the outcomes of which drive breeding and product development. In order for basic, translational, and applied researchers to benefit from others' investigations, newly generated data must be made freely and easily accessible. MaizeGDB is the maize research community's central repository for genetics and genomics information. The overall goals of MaizeGDB are to facilitate access to the outcomes of maize research by integrating new maize data into the database and to support the maize research community by coordinating group activities.

## 1. INTRODUCTION

Maize (Zea *mays* L.) is a species that encompasses the subspecies *mays* (commonly called “corn” in the US) as well as the various teosintes that gave rise to modern maize. Maize is an important crop: not only is it one of the most abundant sources of food and feed for people and livestock the world over, it is also an important component of many industrial products. Maize byproducts are present in, for example, glue, paint, insecticides, toothpaste, rubber tires, rayon, and molded plastics, among others. Maize is also currently the nation's major source of ethanol, a major biofuel that is more environmentally friendly than gasoline and that may be a more economical fuel alternative in the long run. Although it is unlikely that ethanol production from maize directly will be sustainable long-term, maize's suitability to serve as a model organism for developing fuelstock grasses is apparent [1]. Indeed, in addition to its value as a commodity, maize has been a premiere model organism for biological research for over 100 years. Many seminal scientific discoveries have first been shown in maize, such as the identification [2] and cloning [3] of transposable elements, the correlation between cytological and genetic crossing over [4], and the discovery of epigenetic phenomena [5]. These exceptional characteristics of maize set this amazing plant apart: no other species serves as both a commodity and a leading model for basic research.

Today, with the accelerated generation of maize genetic and genomic information, the need for a centralized biological data repository is critical. MaizeGDB (the **Maize** **G**enetics and genomics **D**ata **B**ase [6]) (http://www.maizegdb.org/) is the Model Organism Database (MOD) for maize. Stored at MaizeGDB is comprehensive information on loci (genes and other genetically defined genomic regions including QTL), variations (alleles and other sorts of polymorphisms), stocks, molecular markers and probes, sequences, gene product information, phenotypic images and descriptions, metabolic pathway information, reference data, and contact information for maize researchers. Described in the results and discussion section are example workflows that could be followed by researchers to utilize the MaizeGDB resource for their research. Other long-term resources serving maize data include Gramene (http://www.gramene.org/) [7], which specializes in grass comparative genomics, and GRIN (the Germplasm Resources Information Network; http://www.ars-grin.gov/npgs/), which provides access to the National Plant Germplasm System's germplasm stocks and related breeding data. MaizeGDB makes an effort to guide researchers to these resources via context-sensitive linkages rather than duplicating data, though some data are shared simply to allow for the context-sensitive linkages to be created. This reduces duplication in effort and allows personnel skilled in comparative genomics and germplasm conservation/plant breeding to interact with maize researchers directly via Gramene and GRIN, respectively.

In addition to storing and making maize data available, the MaizeGDB team also provides services to the community of maize researchers and offers technical support for the Maize Genetics Executive Committee and the Annual Maize Genetics Conference. Also available at the MaizeGDB website, as a service to the maize research community, are bulletin boards for news items, information of interest to cooperators, lists of websites for projects that focus on the scientific study of maize, the Editorial Board's recommended reading list, and educational outreach items.

The genetic and genomic data as well as community-related information maintained by MaizeGDB are highly utilized: MaizeGDB averages 8620 visitors (based on unique Internet Protocol or IP addresses) and over 160 000 page impressions per month (July 2007 to June 2008). In addition, MaizeGDB came in fifth out of 170 in a National Plant Genome Initiative Grantees poll in which lead principal investigators reported most useful websites for their research [8].

## 2. MATERIALS AND METHODS

### 2.1. Kinds of data in the database that link genetic and genome sequence information

MaizeGDB is the primary repository for the major genetic and cytogenetic maps and includes details about genes, mutants, QTL (quantitative trait loci), and molecular markers including 2500 RFLPs (restriction fragment length polymorphisms), 4625 SSRs (simple sequence repeats), 363 SNP (single nucleotide polymorphisms), 2500 indels (insertion/deletion sites), and 10 644 overgos (**over**lapping oli**go**nucleotides). These data are described using 1.27 millions synonyms, 42 000 primer sequences, 16 394 raw scores from mapping based upon 16 panels of stocks, and 323 313 links to GenBank [9] accessions. GenBank accessions form the links between the genetic position on a chromosome, the sequence records at MaizeGDB, and the EST (expressed sequence tag) and GSS (genome survey sequence) contig assemblies at PlantGDB [10] and Dana Farber (The Gene Indices at http://compbio.dfci.harvard.edu/tgi/cgi-bin/tgi/gimain.pl?gudb=maize, previously at TIGR [11]). All of the 3 520 247 sequences in MaizeGDB are accessible by BLAST [12] and can be filtered to report only mapped loci, including any SSRs and overgos that may not be mapped genetically, but via BACs (bacterial artificial chromosomes) in anchored contigs.

The inclusion of the public BAC FPC (Finger Print Contig) information [13] adds 439 449 BACs together with associated overgo, SSR, and RFLP markers, which are used to assemble the contigs and to link contigs onto genetic map coordinates. The order of loci on the BAC contigs is represented by over 27 000 sequenced-based loci on the IBM2 FPC057 maps (http://www.maizegdb.org/cgi-bin/displaymapresults.cgi?term=ibm2+fpc0507) in MaizeGDB, by links to contigs at both the Arizona FPC site (http://www.genome.arizona.edu/) and the genome sequencing project (http://www.maizesequence.org/). As the B73 genome sequence progresses, these BAC sequences are added to MaizeGDB along with links to the sequencing project, both from the BAC clones and from genetically mapped loci associated with a BAC.

The newest maps in MaizeGDB, IBM SNP 2007 (http://www.maizegdb.org/cgi-bin/displaymapresults.cgi?term=ibm%20snp%202007), are the first of a new generation of genetic maps from the Maize Diversity Project (http://www.panzea.org/) kindly provided pre-publication by Dr. Mike McMullen. The SNP loci on these maps are associated with allelic sequences from a core set of maize and teosinte germplasm. Because the majority of the anticipated 1128 loci have been previously mapped onto BAC clones [13, 14], these genetic maps tightly link sequence diversity to the B73 genome sequence.

### 2.2. Methods of access, environments, and the database back end

#### 2.2.1. The production web interface

**Figure 1:**
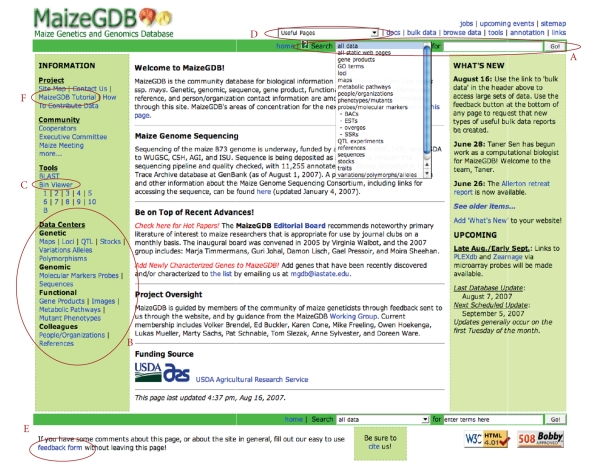
The MaizeGDB home page. The most commonly utilized search functionality for MaizeGDB is the search bar (A), which is available within the header of any MaizeGDB page. To browse data and to search specific data types using specific limiters, the Data Centers (B) are also quite useful. Also available is a Bin Viewer (C), which allows for a view of lots of data types within the context of their chromosomal location. To enable access to the Data Centers and other displays of interest from any MaizeGDB page, a pull-down menu for “Useful pages” (D) is accessible on the header of any MaizeGDB page. The footer of all MaizeGDB pages contains a context-sensitive “feedback form” link (E). Researchers use the feedback form to report errors, ask questions, and to contact the MaizeGDB team directly. For newcomers to the site, the MaizeGDB Tutorial (F) can help them to get a jump start on how to use the site.

Maize researchers primarily access MaizeGDB through the series of interconnected Web pages available at http://www.maizegdb.org/ (see Figure 1). These web pages are dynamically generated and are written in PHP (the recursive abbreviation for PHP Hypertext Preprocessor [15]) and Perl [16]. Through this interface, each page shows detailed information on a specific biological entity (such as a gene) as well as basic information about data associated with it (genes are associated with maps, phenotypes, and citations, among others). These additional data types are linked to the gene page, enabling quick access to alternative data views. The site also includes links to related resources at other databases; genes, for example, are linked to Gramene [7].

**Figure 2:**
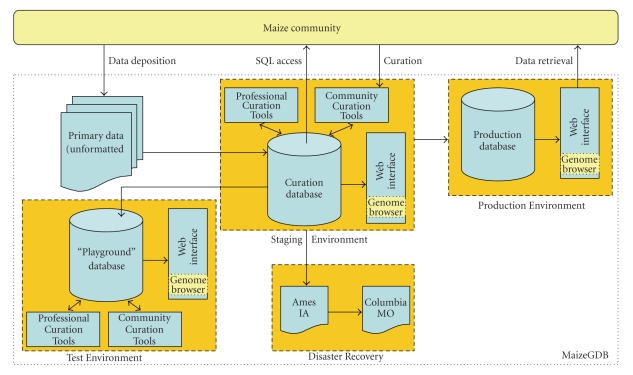
Simplified infrastructure of MaizeGDB. The community of maize researchers can add data to the database (downward-facing arrows from the uppermost yellow box) via direct data deposition (upper left) and via a set of Community Curation Tools that interacts with the Curation Database (upper center). Researchers are also allowed access to maize data (upward-facing arrows from the lower dashed box) via a web interface that can be accessed at http://www.maizegdb.org/ (upper right) and by way of SQL access to the Curation Database, which houses the most up-to-date data available (upper center). These functionalities are supported by two of the three environments: Production and Staging, respectively (upper dashed gold boxes). Available for use by MaizeGDB personnel to facilitate data modeling and trial programming manipulations is a third environment called Test (lower left dashed gold box), which is identical to the Staging Environment. To ensure that the most up-to-date copy of the database is backed up, a Disaster Recovery process has been instituted (lower center dashed gold box) whereby a compressed copy of the database is backed up to a separate machine in Ames, Iowa daily, and to a server in Columbia, Missouri weekly.

One may access these individual data pages by using either (1) the search bar located at the top right of every page (Figure 1(A)), or (2) data type-specific advanced querying tools (accessible via the “Data Centers” links; Figure 1(B)) on the left side of the home page, or (3) the Bin Viewer tool (Figure 1(C)), which is located in the left margin of the home page or via a pull down labeled “Useful pages” (Figure 1(D)) accessible at the top of any MaizeGDB page. These tools allow researchers to easily find relevant data displays.

MaizeGDB's method of data delivery has three primary goals: placing information within the framework of its scientific meaning, making this information available to the researcher with minimal input (often only the relevant term), and requiring minimal effort from the researcher to comprehend the data displays. By focusing on biological context and ease of use as the primary focus of this interface (the “production” Web interface), the database is intended to be intuitive to the researcher as their click stream follows a logical path of biological associations. Up-to-date site usage statistics can be accessed online at http://www.maizegdb.org/usage/.

#### 2.2.2. Structure and relationship of environments: production, staging, and test

The production Web interface, which most MaizeGDB users interact with, is only one component of the overall MaizeGDB infrastructure (Figure 2). The data accessed by the production Web interface are typically updated on the first Tuesday of each month. Prior to being in that Production Environment, the data are prepared for public accessibility in a Staging Environment. In the Staging Environment, the most up-to-date information is available, new data are added to the database, and existing data are updated with new information. In addition to a Web interface that appears identical to the one in the Production Environment, the Staging Environment offers SQL (Structured Query Language) read-only access to the community so that researchers interested in interacting with the data in a more direct and customized manner can have access to the most up-to-date information available. In addition, a Disaster Recovery system has been put in place whereby the Curation Database is backed up in a compressed format to a separate machine in Ames, Iowa daily. Once weekly, the Ames file is copied to Columbia, Missouri for off-site storage.

To aid in the modeling of new types of data for inclusion in the MaizeGDB product and to enable programming to be tried out in a safe place, a Test Environment identical to the Staging Environment has been created. Note that three copies of the database exist. While each environment and server has a specific purpose, all are configured such that they could serve a backup to each other. If any one server was to fail, either of the other two could provide full, unrestricted data access and site functionality. The curation database is backed up on a daily basis and is available for download (http://goblin1.zool.iastate.edu/~oracle/) for those who have Oracle Relational Database Management System (RDBMS) installed locally.

#### 2.2.3. Curation

Also available within the Staging Environment are Community Curation Tools to enable researchers to add small datasets to the database directly, as well as a set of Professional Curation Tools developed by Dr. Marty Sachs' group at the Maize Genetics Cooperation-Stock Center in Urbana-Champaign [17]. Whereas the Community Curation Tools have many safeguards to help researchers enter data step-wise and with enforced field requirements, the Professional Curation Tools allow MaizeGDB project members as well as Stock Center personnel to enter datasets in a more stream-lined and powerful fashion with fewer integrity enforcement rules (which slow down the data entry process considerably). It also should be noted that data added to the database via the Community Curation Tools are first marked as “Experimental” that must be “Activated” by professional curators at MaizeGDB. This ensures that only quality information is made publicly accessible. The availability of a Curation Web interface (within the Staging Environment) enables researchers to view the data as they will appear once they are uploaded to Production. Few researchers (about 30 at present) have Community Curation accounts. To increase the use of these tools, training sessions are being organized (see Section 2.3, below). If researchers wish to deposit complex or large datasets, it would not be reasonable to enter the data via the Community Curation Tools because those tools work via a “bottom-up” approach whereby the records are (1) built based upon the most basic information included in the dataset and (2) entered one record at a time (i.e., not in bulk). For complex or large datasets, researchers are encouraged to submit data files to the curators at MaizeGDB. Those data are added to the database directly by curators and the database administrator.

#### 2.2.4. Database back end

Each environment's server has a perpetual license and is supported by Oracle RDBMS powered by 2 × 2.0 GHz Xeon processors, 4 GB of RAM, 5 × 73 GB Ultra 320 10 K RPM drives with Red Hat Advanced Server 2.1 operating system installed. The curation database, either partially or in its entirety, can be moved to MySQL, Microsoft Access, and nearly any other portable data format that a researcher would need. Requests to gain read-only SQL access to the Curation database can be made via the feedback link that appears at the bottom of any MaizeGDB page. Data housed at MaizeGDB are in the public domain and are freely available for use without a license.

### 2.3. Outreach

One of the strengths of MaizeGDB is its responsiveness to community input, received either personally or by the feedback forms accessible at the bottom of each page (Figure 1(E)). To provide outreach and user support as well as to solicit input from researchers in a more active manner, several strategies are employed. The first is tutorials and basic information on MaizeGDB. The MaizeGDB Tutorial (Figure 1(F)) can be reached from the home page at the top of the left margin. A new user can go through this tutorial, and become familiar with how to use the site quickly. In addition, a “Site Tour” with an overview with examples can be found under the “Useful pages” pull down menu at the top of each page. More specific tutorial examples and other educational materials are available via the “Education” link, also within the “Useful pages” pull down menu. Also, on many of the “Data Center” pages (available from the left margin of the front page or via the “Useful pages” pull down) a discussion of the topic of the page that is suitable for the general public appears toward the bottom. Another form of outreach supported by MaizeGDB is assistance at meetings and conferences. Representatives from MaizeGDB attend and help researchers at the Annual Maize Genetics Conference (usually in March), the International Plant and Animal Genome Conference (January), and various other meetings through direct interaction in person. Finally, researchers can request a MaizeGDB site visit. About three times a year, an expert curator travels to various research locations and provides tutorials and support for maize researchers. For these visits, the local maize researchers are asked for a list of specific questions ahead of time. During the one to two day visits, researchers interact in groups and one-on-one with the traveling curator to learn how to utilize MaizeGDB for their research and to deposit data at MaizeGDB.

### 2.4. Community support services

MaizeGDB provides community support in several ways. Two members of the MaizeGDB team, MLS and TES, serve as ex officio members of the Maize Genetics Conference Steering Committee. They collect electronic abstracts for the Annual Maize Genetics Conference and handle the preparation and printing of the program for the conference. MaizeGDB personnel also manage regular community surveys on behalf of the Maize Genetics Executive Committee. These surveys enable the Executive Committee to summarize the overall research interest of the maize community and to advise funding agencies on future research directions. MaizeGDB personnel also manage the Executive Committee's website (i.e., http://www.maizegdb.org/mgec.php) and conducts the Executive Committee's elections. MaizeGDB houses the mailing list for the annual Maize Newsletter and project personnel conduct semi-regular mailings to the maize community on behalf of interested researchers by maintaining an electronic list of researchers' contact information. Potential mailings to this list are vetted by the Executive Committee.

## 3. RESULTS AND DISCUSSION

**Figure 3:**
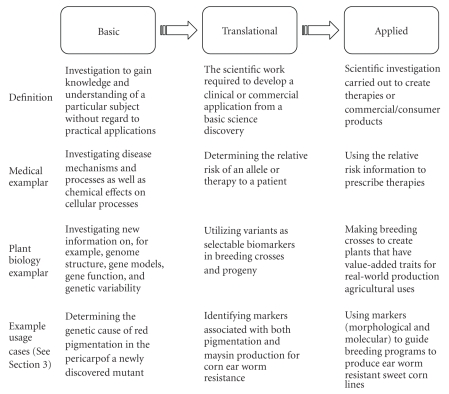
Three types of biological research. Research can be divided into three categories: basic, translational, and applied. Outcomes from basic research feed into translational predictions, and developed uses for these findings constitute the basis for developing real-world applications that benefit humanity and the world. Listed after the flow of research are definitions for each research type as well as medical and plant biological models for how the different divisions are interrelated. Also shown are overviews of the example usage cases presented in Section 3.

To demonstrate how researchers utilize MaizeGDB, three example usage cases are presented here. Because researchers with very different goals can all utilize MaizeGDB to advance their work, the usage cases are classified by research type: basic, translational, and applied. See Figure 3 for examples of how these research types fit together. By enabling researchers to carry out workflows that support translational and applied research, MaizeGDB plays a part in influencing crop development directly. Although a single researcher might even include all of these three aspects in his/her research simultaneously, here the researcher types are distinguished as follows: basic researchers investigate the fundamental biology of the organism, translational researchers work to determine the application of basic research outcomes for practical purposes [18], and applied researchers implement proven technologies to improve crops.

### 3.1. Basic

Many basic researchers work with mutants to understand the processes underlying biological phenomena. Once a new mutant is found, there are several standard methods used to elucidate normal gene functions. These efforts include determining whether the mutant represents an allele of a previously described gene, and if not, genetic mapping and cloning of the new gene. Information stored in MaizeGDB is useful in all of these steps.

In a large screen for mutations that change pericarp pigmentation from red to some other color, Researcher 1 has found a plant with a brownish-red pericarp coloration. She first wants search MaizeGDB to find all known mutants that have red pericarp phenotypes to determine whether this mutation represents a newly discovered gene. Because she does not know how others might have described the phenotype, she decides to browse existing phenotype terms and images. From the left margin of the MaizeGDB homepage, she selects “Mutant Phenotypes” under “Data Centers-Functional.” On this page (http://www.maizegdb.org/), she selects “pericarp color” from the pull down menu labeled “Show only phenotypes relating to this trait” in the green search bar. A number of possible mutant phenotypes are returned, including “red pericarp.” Clicking on the “red pericarp” phenotype link, she finds that the listed mutants are alleles of *p1* (*pericarp color1*). On this page (http://www.maizegdb.org/cgi-bin/displayphenorecord.cgi?id=13818), she scrolls to the bottom and finds that there are many stocks that can be ordered from the Maize Genetics Cooperation-Stock Center that carry *P1-rr* (an allele that causes red pericarp and red cob) or *P1-rw* (red pericarp and white cob). Having these stocks in hand will enable her to test whether the new mutant represents an allele of the *p1* gene, so she decides to order a few for complementation analyses. Clicking on the stock links listed on the variation/allele page allows her access to a shopping cart utility (in the green right hand panel), and she orders seed from the Stock Center directly through the MaizeGDB interface. She then goes back to the results of her “pericarp color” query and repeats the process for “cherry pericarp,” ordering stocks for *r1-ch* (*colored1-cherry*), also to be used in her complementation analyses. (Another way she could have found maize stocks that have red pericarp is the following: from the header of any page, select “Useful pages” and click “Stocks.” This pulls up the stock search page http://www.maizegdb.org/stock.php. In the green box, select stocks with the phenotype “red pericarp” from the pull down menu of all phenotype names and submit. A long list of stocks that contain alleles of *p1* with red pericarp is returned. Alternatively, the Stock Center Catalog is also available from the Stocks Data Center page.)

Researcher 1 receives several appropriate stocks and performs allelism tests and determines that her mutant (which turns out to be recessive) is not allelic to *p1* or *r1.* She returns to MaizeGDB and again looks through “Mutant Phenotype” results using the “pericarp color” query. Listed there are brown pericarp, orange pericarp, white pericarp, and lacquer red pericarp phenotypes in addition to the red and cherry phenotypes she focused on initially. She finds that there is no stock available for the brown pericarp phenotype (the *brown pericarp1* mutant has been lost), and all the others are alleles that confer colored pericarp in the dominant condition as a result of the presence of *P1* alleles. To determine whether the new mutation could be an allele of *bp1*, she decides to map it genetically.

MaizeGDB houses the largest collection of publicly available genetic maps of maize (currently over 1,337 maps). These include maps of genes primarily defined by mutants with morphological phenotypes (“Genetic 2005” is the most current), maps based on phenotypic molecular markers, and composite maps where various maps have been integrated. These maps can be easily accessed from the home page, via the left margin link to “Data Centers-Genetic-Maps” (http://www.maizegdb.org/map.php). This page not only allows various map search functions, but also provides information on the most popular maps and a handy reference to explain more about the various composite maps.

The maize genome is divided into genetic bins of approximately 20 centiMorgans each and boundary markers with nearby SSRs can be used for mapping (for further explanation see http://www.maizegdb.org/cgi-bin/bin_viewer.cgi). Researcher 1 decides to utilize SSRs to map her gene to bin resolution. To find the core markers from the home page, she clicks on “Tools-Bin Viewer” in the left margin of the home page. This provides a list of the core bin markers and a link to purchase relevant primers to screen her mapping population. She generates a mapping population, performs PCR experiments using the polymorphic markers, and maps her mutant to bin 9.02.

To see what genes are located in bin 9.02, she goes back to the Bin Viewer (from the homepage), and holds the curser over the image of chromosome 9 until she sees “bin 9.02,” then clicks. The result is a long list of genes, other loci, sequences, EST contigs, SSRs, BACs, and other data relating to bin 9.02. Searching through this data, she sees that *bp1* is listed under “other loci” in bin 9.02. This is a “lapsed locus” meaning that the stock has been lost, but perhaps she has found a new allele!

To see more specific genetic mapping data on *bp1*, she goes to the search bar along the top green bar of every page, selects “loci” from the pull down menu, types “bp1” into the field provided, and clicks the button marked “Go!” This brings her to the *bp1* locus page (http://www.maizegdb.org/cgi-bin/displaylocusrecord.cgi?id=61563) where she can see that *bp1* is placed on three genetic maps. Clicking on each map, Researcher 1 learns that in 1935, *bp1* was mapped between *sh1* and *wx1* (*shrunken1* and *waxy1*), two well-studied genes. To search for molecular markers suitable for fine structure mapping, she visits “Data Centers-Genetic-Maps” from the link on the home page. In the green Advanced Search box, she enters *sh1* and *wx1* separately in the “Show only maps containing this locus” lines. This returns only genetic maps that contain both genes. She selects the map with the most markers—IBM2 2005 Neighbors 9 (with 2,488 markers). She finds *sh1* at position 80.30, and *wx1* at 185.00. To choose among several molecular markers, Researcher 1 follows the available links leading her to information about suitable primers, a number of variations (which can help to decide if there may be a polymorphism in her mapping populations), gel patterns, and any available GenBank accession numbers for sequences as well as sequenced BACs. She finally selects markers and performs fine structure mapping. As she finds markers closer and closer to the gene, she can proceed with positional cloning to determine whether the position is consistent with *bp1* (nice examples of how this is done can be found in [19–21]).

### 3.2. Translational

Research to understand the metabolic pathways that produce pigmentation (like those outlined in Section 3.1) are well studied in maize [22]. One example of a well-characterized gene that confers pigmentation is *p1*, which encodes a transcription factor that regulates synthesis of flavones such as anthocyanins [23]. The *p1* gene, along with its adjacent duplicate *pericarp color2* (*p2*), controls pericarp and cob coloration and causes silks to brown when cut. One flavone produced by the pathway is maysin, a compound which has been shown to be antinutritive to the corn ear worm at concentrations above 0.2% fresh weight if husks limit access to the ear such that feeding on silks is required for the insect to enter [24]. Many QTL for resistance to corn earworm map near loci in the flavone synthesis pathway that are either regulatory genes (such as *p1* and *p2*), or at rate-limiting enzymatic steps, such as *c1* (*chalcone synthase1*) that contribute maysin accumulation in silks [25]. Understanding how maysin functions and how this information could be used for production agriculture is Researcher 2's area of expertise.

Researcher 2 has investigated maysin synthesis for some time, and has decided to clone an uncharacterized maysin QTL near *umc105a*, in the bin 9.02, which is bounded by *bz1* and *wx1* [24]. He believes that the QTL may be a previously described, but lost, *bp1* mutant thought to be involved in maysin synthesis. In the first step, he must first find molecular markers to more finely map the region (his preference would be to use SSRs, since members of the lab are already using them successfully). He plans to follow the strategy of chromosome walking to narrow down the region of interest [19–21] followed by association mapping to identify the actual QTL sequence [26, 27]. Knowing this sequence would enable plant breeders to track the QTL for marker assisted selection.

To find SSR data for mapping to a bin region, Researcher 2 goes to the MaizeGDB home page and clicks on “Data Centers-Genomic-Molecular Markers/Probes” in the left margin, then clicks the “SSR” link at the top of the page (the link is located in *“Specific information is available on BACs, ESTs, overgos, and SSRs*.”) Scrolling down to the green “Set Up Criteria” box, he then selects bin 9.02 and submits a search request. A report is returned that lists the available SSRs for bin 9.02, complete with primers, gel patterns for different germplasm, and related maps. By going back to the SSR page, he also downloads tabular reports of map locations of all SSRs on chromosome 9, including those that have been anchored to a BAC contig. Using this information in the laboratory, members of his research group perform mapping experiments using several SSRs in bin 9.02 along with some others in the more distal part of bin 9.03. They discover that the mid-region peak for the QTL is very near an SSR for *bnlg1372*, which is anchored to a BAC contig.

To find sequenced BACs that may harbor the earworm resistance QTL, Researcher 2 uses the search bar at the top of each MaizeGDB page to find the locus *bnlg1372.* At the top of the *bnlg1372* page, he follows a link to the contig 373 display at the Maize Sequencing Project site (http://www.maizesequence.org/). This is a rather large contig with many sequenced BACs and assigned markers. At the Maize Sequencing Project site, he uses the export function (a button at the left margin) to view a text list of all the markers and sequenced BAC clones that are available on the Finger Print Contig physical map. He finds that *bnlg1372* is assigned to the region “19742100,1974700,” encompassed by the sequenced BAC clone, c0324E10. This information provides coordinates for viewing the region on a large contig associated with *bnlg1372*, the sequence of BAC c0324E10, and any other BACs nearby. Researcher 2 sequences candidate regions in diverse germplasm and conducts association analysis using silk maysin levels as a trait. This may require other information about nearby markers, which also are accessible via MaizeGDB [28, 29].

Although these investigations may require the development of further sequenced-based markers, Researcher 2 hopes that useful markers already exist and decides to explore MaizeGDB for any other sequences or primer-based markers already assigned to the region of interest including SNPs and indels. To do this from the locus page for *bnlg1372*, he clicks on the link to the most current IBM neighbors map listed, then explores the “sequence” and “primer” view versions of the map by clicking on the relevant links at the top of the page just under the map name. The primer view shows primers associated with mapping probes along with the name of the probes—just what he needs to get going with the association mapping work.

### 3.3. Applied

Interested in breeding plants for organic sweet corn production, Researcher 3 has decided to use molecular markers to select for high maysin content, which would increase resistance to the corn earworm—a cause of significant damage to sweet corn [30]. Although plants could be genetically modified to carry the genes that confer high maysin levels in silks (e.g., see [31]), Researcher 3's farming clients require that their product be certified as both organic and “GMO-free.” To meet the producers' needs, he has decided to pursue a marker-assisted selection program to create high maysin sweet inbred lines, which he will use to generate single-cross hybrids. To get started with the work, he searches MaizeGDB to find references, markers, and stocks for the project. Described here are the details on how he could use MaizeGDB to (1) access stocks known to have high maysin content directly and (2) locate relevant stocks based upon associated data with no prior knowledge of which stocks he wants to find. An outline of how he uses MaizeGDB to identify relevant selectable markers for tracking the various QTL associated with maysin accumulation also is described.

In the instance of looking for particular stocks, Researcher 3 has identified GT114 as a high maysin line from [25]. Using the green search bar at the top of any MaizeGDB page, he searches “stocks” for “GT114.” At that page, he sees a brief annotation stating that GT114 is a poor pollen producer and makes a note of that observation and plans to cross by IA453 and IA5125, sweet lines that produce pollen well, to ameliorate this potential difficulty. Clicking the link to GT114, he sees that it is an inbred line derived from GT-DDSA (DD Syn A) in Georgia, and it is made available via GRIN. Selecting the link for GRIN, a page opens at that site (http://www.ars-grin.gov/cgi-bin/npgs/html/search.pl?PI+511314). Listed there are the *Crop Science* Registration data, availability (noted as currently unavailable, but a call to Mark Millard, maize curator at the maintenance site indicates that he could access that stock in limited quantities if current resources allow), and an image of bulk kernels among other information. The image of bulked kernels is especially revealing: the kernels are yellow and the cob fragments appear red. Aware that a red cob would be unacceptable for breeding sweet corn (the red pigment could cause quite a mess for those cooking and eating corn on then cob), he decides to search MaizeGDB for other available high maysin stocks.

After a literature search of breeding stocks with a white cob that might still produce maysin in the silks, Researcher 3 starts searching stocks for those known to carry the *P1-wwb* allele, a dominant allele of the *p1* locus that confers white pericarp, white cob, and browning silks. By clicking the “Data Centers-Genetic-Stocks” link from the MaizeGDB homepage, he arrives at the Stocks Data Center page (which is also accessible via the “Useful pages” pull down at the top of every MaizeGDB page). He uses the Advanced Search box to limit the query by variation to those stocks associated with the allele *P1-wwb*. A number of the stocks returned on the results page have been evaluated for silk maysin accumulation (per associated publications) and could be further investigated as potential breeding stocks.

Although the *p1* gene accounts for much of the variability in maysin accumulation [32], association and QTL analyses for candidate genes for maysin accumulation also have identified *anthocyaninless1* (*a1*), *colorless2* (*c2*), and *white pollen1* (*whp1*) as contributing significantly [32, 33]. Researcher 3 can track the dominant *P1-wwb* allele visually by selecting for browning silks given that the sweet lines he will be using in the breeding program have silks that do not brown, but tracking the other factors will require the use of molecular markers. To find molecular markers to select for desirable alleles of, for example, *a1*, Researcher 3 uses the search menu at the top of any page at MaizeGDB to find “loci” using the query “a1.” The results page (http://www.maizegdb.org/cgi-bin/displaylocusresults.cgi?term=a1) lists many loci with a1 as a substring, but shows the exact match (the *a1* locus) at the top of the list. Clicking on that link shows the *a1* locus page (http://www.maizegdb.org/cgi-bin/displaylocusrecord.cgi?id=12000), which lists useful information including six probes/molecular markers that could be used for tracking useful *a1* alleles. Using the same process, he also finds markers for the *c2* and *whp1* loci and sets to work determining which markers to use for his selections.

## 4. CONCLUSIONS

Because MaizeGDB stores and makes accessible data of use for a variety of applications, it is a resource of interest to maize researchers spanning many disciplines. The fact that basic research outcomes are tied to translational and applied data enables all researcher types to utilize the MaizeGDB resource to further their research goals, and connections to external resources like Gramene, NCBI, and GRIN make it possible for researchers to find relevant resources quickly, irrespective of storage location.

At present, maize geneticists are at the cusp of a milestone: the genome of the maize inbred B73 is being sequenced in the U.S., with anticipated completion in 2008. In addition, scientists working in Mexico at Langebio (the National Genomics for Biodiversity Laboratory) and Cinvestav (Centro de Investigacion y Estudios Avanzados) have announced through a press release (July 12, 2007) that they completely sequenced 95% of the genes with 4X coverage in a native Mexican popcorn called palomero, though the data have not yet been released and the quality of the data is unknown (see http://www.bloomberg.com/apps/news?pid=20601086&sid=aO.Xj8ybAExI&refer=latin_america). At present and as more maize sequence becomes available relating sequences to the *existing* compendium of maize data is the primary need that must be met for maize researchers in the immediate future. Creating and conserving relationships among the data will enable researchers to ask and answer questions about the structure and function of the maize genome that previously could not be addressed. To address this need, MaizeGDB personnel will create a “genome view” by adopting and customizing a Genome Browser that could be used to integrate the outcomes of the Maize Genome Sequencing Project. For genome browser functionality, basic researchers have an interest in visualizing genome structure, gene models, functional data, and genetic variability. Translational researchers would like to be able to assign values to genomic and genetic variants (e.g., the value of a particular allele in a given population) and to view those values within a genomic context. Applied researchers are interested in tagging variants for use as selectable markers and retrieving tags for particular regions of the genome. To best meet these researchers' needs, the “genome view” will allow researchers to visualize a gene within its genomic context and a soon to be created “pathway view” will enable the visualization of a gene product within the context of relevant metabolic pathways annotated with Plant Ontology (http://www.plantontology.org/) [34] and Gene Ontology (http://www.geneontology.org/index.shtml) [35] terms. By making sequence information more easily accessible and fully integrated with other data stored at MaizeGDB, it will become possible for researchers to begin to investigate how sequence relates to the architecture of the maize chromosome complement. How are the chromosomes arranged? Is it possible to relate the genetic and cytological maps to the assembled genome sequence? Are there sequences present at centromeres that signal the cell to construct kinetochores, the machines that ensure proper chromosome segregation to occur, at the correct site? MaizeGDB aims to enable researchers to discover answers to such queries that will enhance the quality of basic maize research and ultimately the value of maize as a crop. It will become possible to interrogate the database to find answers to these and other complex questions, and the content of the genome can better be related to its function, both within the cell and to the plant as a whole. Convergence of traditional biological investigation with the knowledge of genome content and organization is currently lacking, and is a new area of research that will open up once a complete genome sequence and a method for searching through the whole of the data are both in place. It is the ability to investigate and answer such basic research questions that will serve as the basis for devising sound methods to breed better plants. Once the relationships among sequence data and more traditional maize data like genotypes, phenotypes, stocks, and so forth have been captured, it is important that those data be presented to researchers in a way that can be easily understood without requiring that they have any awareness of how the data are actually stored within a database. It is these needs—creating connections between sequence and traditional genetic data, improving the interface to those data, and determining how sequence data relate to the overall architecture of the maize chromosome complement—that the MaizeGDB team seeks to fulfill in the very near future.
